# Technical Complications Associated with Embolic Protection Device During Carotid Artery Stenting: Incidence, Risk Factors, Clinical Implications, and Rescue Maneuvers

**DOI:** 10.3390/diagnostics14232622

**Published:** 2024-11-21

**Authors:** Bo Kyu Kim, Byungjun Kim, Sung-Hye You, Inseon Ryoo, Hye Na Jung

**Affiliations:** 1 Department of Radiology, Korea University Anam Hospital, Korea University College of Medicine, 73, Goryeodae-ro, Seongbuk-gu, Seoul 02841, Republic of Korea; stingray0379@naver.com (B.K.K.); nipponica85@gmail.com (S.-H.Y.); 2 Department of Radiology, Korea University Guro Hospital, Seoul 08308, Republic of Korea; isryoo@gmail.com (I.R.); jhyena@gmail.com (H.N.J.)

**Keywords:** carotid artery stenting, risk management, stroke

## Abstract

**Background/Objectives:** This study aimed to evaluate the incidence, risk factors, clinical implications, and rescue maneuvers of technical complications related to embolic protection devices (EPDs) during carotid artery stenting (CAS). **Materials and Methods:** We retrospectively reviewed all patients who had undergone CAS with EPDs between April 2018 and March 2024. The incidence and types of technical complication associated with EPDs were assessed. Clinical, angiographical, and procedural factors were analyzed to identify risk factors for the occurrence of EPD-related adverse events. Various rescue techniques for managing adverse events were investigated based on the procedure record. **Results:** Of the 158 enrolled patients, the rate of EPD-related technical complications was 23.4% (*n* = 37). Among them, complicated filter retrieval was the most common adverse event (*n* = 23, 14.6%). Older age, a higher degree of residual stenosis, and the type of the EPD were significant risk factors for complicated filter retrieval (*p* < 0.05). Although distal thrombus migration requiring thrombectomy was more frequent in patients with complicated filter removal (2.2% vs. 13.0%, *p* = 0.041), there was no significant increase in postprocedural thromboembolic and hemorrhagic complications. When complicated filter retrieval occurred, careful to-and-fro movement of the patients’ neck, such as rotation, or asking them to swallow was tried first in all 23 patients. When these attempts failed, manipulation of a curved-tip guiding catheter, the balloon bridge technique, and alternative use of a 5 Fr angiocatheter as a retrieval sheath were sequentially tried, and all filters were successfully retrieved. **Conclusions:** Complicated filter retrieval was the most common technical complication during CAS. Various rescue techniques for successful filter removal were effective for ensuring safety of CAS.

## 1. Introduction

Carotid artery stenting (CAS) is a well-established alternative treatment option for carotid endarterectomy (CEA) to prevent stroke in patients with high-grade symptomatic or asymptomatic carotid artery stenosis [1]. Nevertheless, despite equivalent treatment efficacy, thromboembolic complications during or after stenting have been highlighted as limitations regarding the safety of CAS [2]. To minimize the risk of procedure-related stroke, deployment of an embolic protection device (EPD) is recommended during CAS [2,3]. A systematic review including both symptomatic and asymptomatic carotid stenosis cases concluded that using an EPD during CAS resulted in a significantly lower combined periprocedural stroke and death rate (1.8% vs. 5.5%, *p* < 0.001) [4]. Specifically, in patients with a high risk of surgical complications, the risk associated with CAS in conjunction with an EPD may be lower than that associated with CEA [2].

An EPD consists of a filter that can capture emboli while maintaining blood flow and a wire on which the filter is mounted. Although CAS with an EPD is technically successful in most cases, several untoward events related to protection devices have been reported [5]. Undesirable events associated with protection devices may occur during any procedural step of CAS and include unsuccessful deployment of the filter, filter wedging into the stent delivery catheter tip, arterial dissection, flow-limiting spasm, flow impairment due to clogging of large amounts of embolic material, and detainment of the filter [6,7]. Therefore, preparing appropriate countermeasures against these events in advance is essential to increase technical success rates and ensure safety during CAS procedures. Therefore, we investigated the incidence of EPD-related adverse events and describe herein our experiences with rescue techniques.

## 2. Material and Methods

### 2.1. Study Population

We retrospectively reviewed the data of both symptomatic and asymptomatic patients who had undergone CAS at our institution between April 2018 and March 2024. Patients who were identified as having carotid artery stenosis of 50% or more upon non-invasive vascular study underwent digital subtraction angiography (DSA) for further evaluation. Among these patients, those who met the following criteria were treated with CAS: (1) symptomatic patients with stenosis of 50% or more, (2) asymptomatic patients with stenosis of 70% or more, (3) patients with more than 50% stenosis and contralateral carotid occlusion, (4) symptomatic patients with carotid dissection, or (5) patients with acute ischemic symptoms (within 24 h) and a National Institutes of Health Stroke Scale (NIHSS) score of ≥4 due to severe stenosis or occlusion of the internal carotid artery (ICA). Patients were classified as symptomatic if they had a history of transient ischemic attack, amaurosis fugax, or minor nondisabling stroke. Patients who underwent CAS without the use of distal cerebral protection were excluded from this study.

### 2.2. Procedures

All procedures were performed via a transfemoral approach. An 8 Fr guiding catheter (VISTA BRITE TIP^®^ Guiding Catheter; Cordis Corporation, Miami, FL, USA) with a straight or curved tip and 100 cm in length was placed in the distal common carotid artery. Another 8 Fr catheter with a curved tip and 80 cm in length (Guider Softip; Boston Scientific, Maple Grove, MN, USA) was alternatively used when navigation difficulty was expected due to severe angulation at the carotid bulb. Cerebral protection was achieved using either Emboshield NAV (Abbott Vascular, Santa Clara, CA, USA) or SpiderFX (Medtronic, Plymouth, MN, USA). The EPD was carefully advanced using a mounted filter wire under fluoroscopic guidance to pass through the stenotic segment and placed at a relatively straight segment of the distal cervical ICA. Initial angioplasty was performed using a 3 to 4 mm monorail-type balloon over a 0.014 filter wire before stent placement. If the residual stenosis was greater than 20%, definite angioplasty was performed using a 4 to 6 mm balloon catheter. A self-expandable stent of open-cell types, such as Acculink (Abbott Vascular, Santa Clara, CA, USA), Protégé (Medtronic, Plymouth, MN, USA), and Precise (Cordis, Miami, FL, USA), or of closed-cell type, such as Carotid Wallstent (Boston Scientific, Galway, Ireland) was deployed across the stenotic segment. After confirming no significant stenosis or in-stent thrombosis, removal of the EPD was attempted using the provided retrieval sheath. The retrieval sheath was carefully advanced along the filter wire while paying attention to the caudal movement of the EPD.

### 2.3. Data Collection

Baseline clinical data, including underlying diseases and antiplatelet therapy within 48 h prior to CAS, were assessed. On DSA, the degree of stenosis was calculated using the North American Symptomatic Carotid Endarterectomy Trial (NASCET) criteria [8]. Near occlusion was defined as two or more of the following: (1) delayed flow in the distal ICA, (2) intracranial collaterals, (3) the ipsilateral distal ICA less than the contralateral distal ICA, and (4) the ipsilateral distal ICA equal to or less than the ipsilateral ECA [9]. The tortuosity index (TI) was defined as the sum of all angles in the stent-placed segment (Supplementary Figure S1).

Technical complications during CAS were classified into three categories of adverse events associated with balloon angioplasty, EPD, and stenting. Balloon angioplasty-related adverse events included severe vasovagal reaction (i.e., sustained hypotension and bradycardia retractable to medication), vascular dissection, and perforation. Adverse events related to the EPD such as unsuccessful deployment of the filter, filter wedging into the stent delivery catheter tip or distal marker of the deployed stent, arterial dissection, flow impairment due to clogging emboli at the filter, complicated retrieval of the filter, and distal thrombus migration after filter removal were evaluated. Complicated filter retrieval was defined as a case where the advancement of the retrieval sheath over the mounted filter wire was not achieved due to resistance during the passage of the retrieval sheath through the stent, requiring additional rescue techniques. Finally, adverse events associated with stent placement encompassed stent migration and in-stent thrombosis.

Postprocedural thromboembolic and hemorrhagic complications were assessed based on the follow-up diffusion-weighted imaging (DWI) and gradient echo images obtained within 24 h after the procedure. Minor infarction included any newly developed diffusion restriction lesions on postprocedural DWI with or without worsening of neurologic symptoms by 3 points according to the National Institutes of Health Stroke Scale (NIHSS) during hospitalization. Major infarction was defined as neurologic deterioration based on worsening of the NIHSS score by 4 or more. Mortality was evaluated until 3 months after the procedure.

### 2.4. Statistical Analysis

Statistical analyses were performed using IBM SPSS Statistics for Windows, version 22.0 (IBM Corp., Armonk, NY, USA). All data are presented as means ± standard deviation or numbers with percentages. Univariate analysis was performed using Student’s *t*-tests for continuous variables and chi-square or Fisher’s exact tests for categorical variables. *p*-Value < 0.05 was considered statistically significant.

## 3. Result

### 3.1. Incidence, Types, and Risk Factors of Intraprocedural Complications

**Figure 1:**
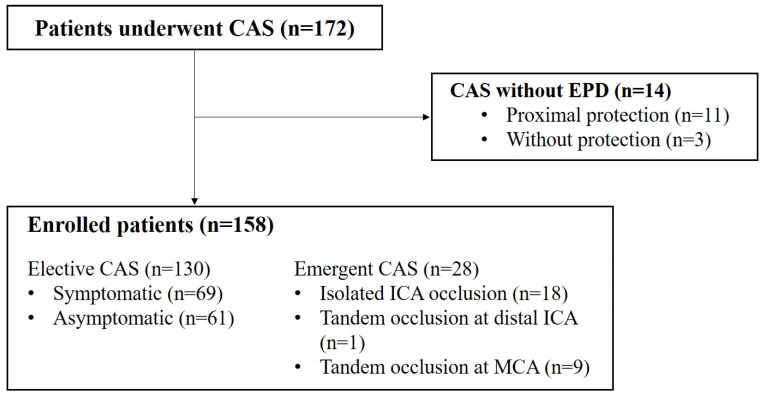
Flow chart of patients included in study. CAS: carotid artery stenting; EPD: embolic protection device; ICA: internal carotid artery; MCA: middle cerebral artery.

Of the 172 patients who underwent CAS, 14 patients were excluded from the study because the EPD was not used during the procedure (Figure 1). In eleven patients presenting with acute occlusion and tight stenosis in the ICA, we were unable to navigate through the stenotic segment using a microwire. For these cases, forceful wire navigation using a 0.035 guidewire was performed, followed by balloon angioplasty under proximal protection using a balloon guiding catheter instead of distal cerebral protection. Additionally, three patients with severely angulated vascular anatomy in the ICA, which did not allow for stable placement of the guiding catheter nor the EPD, were treated with CAS without cerebral protection.

A total of 158 patients were enrolled in this study, and the patient demographics, procedural details, used device, and periprocedural complications are summarized in Table 1. Intraprocedural complications were observed in 40 patients (25.3%), and postprocedural thromboembolic and hemorrhagic complications occurred in 49 (31.0%) and 1 (0.6%) patient, respectively. Three patients (1.9%) with postprocedural major infarction showed aggravation of pre-existing neurologic symptoms even after successful recanalization through emergency CAS. One patient (0.6%) showed an intracerebral hemorrhage in the basal ganglia and frontal lobe 1 day after the procedure, which is a hyperperfusion syndrome. None of the patients died within 3 months.

Among the intraprocedural complications, EPD-related adverse events showed the highest rate of occurrence (*n* = 37, 92.5%). The most common adverse event was complicated retrieval of the filter (*n* = 23, 57.5%), followed by flow impairment due to clogging emboli at the filter (*n* = 14, 35.0%) and distal thrombus migration requiring thrombectomy (*n* = 6, 15.0%). Five patients (*n* = 5, 3.4%) experienced two or more events, of which all five had complicated filter retrieval in common (Table 2). Among the 23 patients who experienced complicated filter removal, 15 patients exhibited impingement of the retriever sheath inside the stent, while the remaining 8 patients displayed impingement at the proximal marker of the stent. Patients who experienced complicated filter removal were of older age, had higher degrees of residual stenosis, and had higher rates of using the Emboshield device (Table 3). Vascular tortuosity measured by the TI and the types of stents did not show significant differences between the two groups. Although the incidence of thrombus migration requiring thrombectomy was higher in patients with complicated filter retrieval (2.2% vs. 13.0%, *p* = 0.041), postprocedural thromboembolic complications such as minor and major stroke did not show significant differences between both groups.

### 3.2. Rescue Techniques for Adverse Events Related to EPD

In all 14 cases with flow impairment due to clogging emboli at the filter, spontaneous resolution of sluggish or blocked antegrade flow could be obtained immediately after filter removal. Upon noticing flow limitation in angiography after stenting, we performed prompt retrieval of the filter, and then the trapped emboli in the retrieved filter were confirmed on visual inspection. However, in two patients, flow impairment and complicated filter retrieval occurred simultaneously, leading to delayed restoration of blood flow and minor infarction. One patient with superimposed distal thrombus migration experienced neurologic deterioration due to the progression of cerebral edema after the procedure, despite successful recanalization.

In all 23 patients with complicated filter retrieval, the filter was successfully retrieved using various rescue techniques. The devices, rescue techniques, and clinical outcomes of the patients are summarized in Supplementary Table S1. When the retrieval sheath could not advance due to being trapped by the stent strut, we typically asked the patient to swallow or rotate their neck, with or without gentle neck compression. Geometric changes between the filter wire and the placed stent were monitored by real-time fluoroscopy, and these maneuvers were performed with sufficient care to ensure that the filter did not move. Nine patients (39.1%) showed successful advancement of the retrieval sheath by using this maneuver.

**Figure 2:**
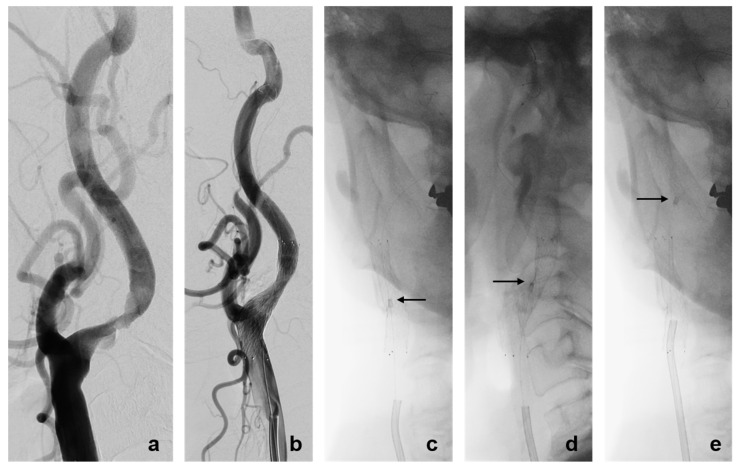
(**a**) Right common carotid angiogram showing focal severe stenosis at the proximal cervical internal carotid artery. (**b**) After carotid stenting with angioplasty, residual mild stenosis was observed. (**c**,**d**) Anterior–posterior and lateral fluoroscopic images during the procedure show that the retrieval sheath could not advance through the stenotic portion. The retrieval sheath advanced toward the anterolateral wall of the internal carotid artery and was prevented from advancing by the stent strut. (**e**) By advancing the guiding catheter with a curved tip, the direction of the retrieval sheath was changed and it could then pass through the stent (arrow).

When patients showed densely calcified plaque and a tortuous carotid bulb on pre-treatment DSA, we employed another curved-tip 8 Fr guiding catheter specifically designed for coiling, such as 80 cm Guider Softip. When the retrieval sheath could not advance due to interference with the protruding stent strut, the guiding catheter was advanced into the proximal stent portion and rotated to change the direction of the filter wire. This allowed the retrieval sheath to pass through the stent more easily so we could safely remove the filter (Figure 2). However, manipulation of the Guider Softip was helpful for changing the direction of the filter wire in only one out of a total of seven trials.

**Figure 3:**
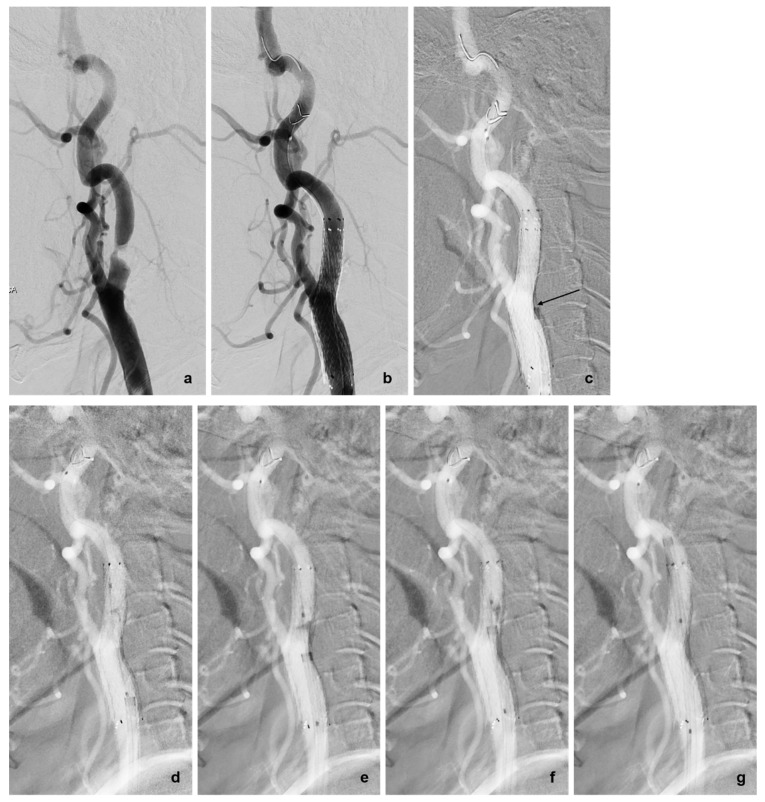
(**a**) Pre-treatment left common carotid angiography showing focal severe stenosis in the proximal cervical internal carotid artery (ICA). (**b**) Carotid angioplasty and stenting were performed under the protection of a 5 mm Emboshield NAV™ embolic protection device (EPD, Abbott Vascular, Santa Clara, CA, USA) in the distal cervical ICA. (**c**) However, the retrieval sheath could not pass through the stent-placed ICA where the filter wire approximated the stent struts presumed to protrude into the lumen of the vessel (arrow). (**d**) A 6 × 30 mm Sterling balloon catheter (Boston Scientific, Natick, MA, USA) was placed in the mid-segment of the stent. (**e**,**f**) After rescue angioplasty, the balloon catheter was left with suboptimal inflation to eliminate the gap between the filter wire and the lumen of the guiding catheter. A guiding catheter was smoothly advanced over the partially inflated balloon catheter. (**g**) After complete bypass of the guiding catheter over the stent-placed carotid bulb, the EPD could be easily and safely removed with the provided retrieval sheath.

**Figure 4:**
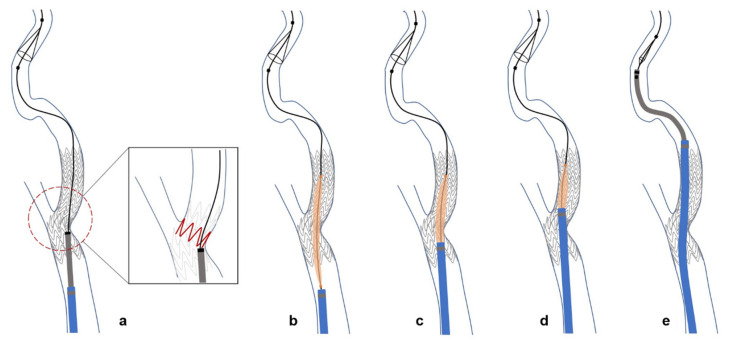
A schematic diagram of a rescue technique with a guiding catheter advanced over a partially inflated balloon catheter. (**a**) Emboshield NAV™ retrieval sheaths are frequently stuck in the intraluminally protruded stent strut at the proximal ICA. (**b**) After placing a balloon catheter in the problematic segment, rescue angioplasty is performed to push a stent strut. Subsequently, the balloon is maintained at a suboptimal pressure of less than 2 atm. (**c**–**e**) If partially inflated, the balloon moves the filter wire away from the stent and eliminates the gap between the inner lumen of the guiding catheter and the filter wire; the guiding catheter can then pass over the problematic segment. After bypassing the stent with the guiding catheter, the retrieval sheath can easily remove the filter.

For patients who did not respond to these maneuvers, we used an adjunctive device, such as a balloon catheter, as a coaxial system to advance the guiding catheter through the deployed stent. A Sterling balloon catheter (Boston Scientific, Natick, MA, USA) was advanced over the wire and placed in the problematic segment of the stent. The balloon was inflated to its nominal pressure, performing angioplasty to detach the filter wire from the protruding stent strut. Subsequently, the balloon was deflated to less than 2 atm pressure to eliminate the ledge effect between the guiding and balloon catheters, allowing the guiding catheter to advance over the partially inflated balloon catheter. As the balloon entered the catheter, it shrank slightly, eliminating the gap between the guiding catheter and the wire without causing balloon rupture. Once the guiding catheter passed through the stent, the balloon catheter was removed. The retrieval sheath was then safely navigated through the bypassed guiding catheter, allowing for successful removal of the filter in seven cases (30.4%) (Figure 3 and Figure 4).

**Figure 5:**
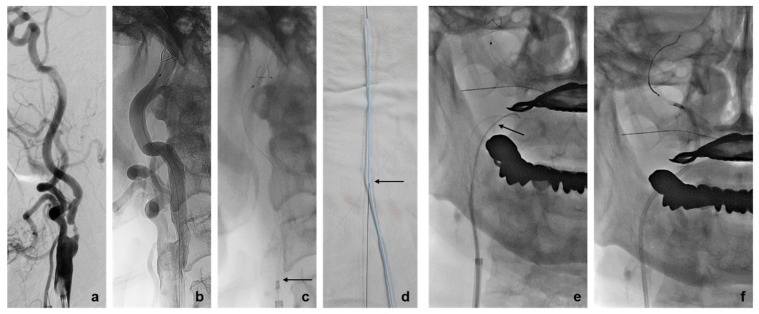
(**a**) Pre-treatment right common carotid artery angiography shows an atherosclerotic ulcerative plaque with focal severe stenosis at the proximal cervical internal carotid artery. (**b**) After carotid angioplasty and stenting, residual mild stenosis occurred due to the calcified plaque. (**c**) The retrieval sheath could not advance over the filter wire at the proximal tip of the stent (arrow). (**d**) A 5 Fr curved-tip angiocatheter was used instead of a retrieval sheath. We created a side hole with a 22-gauge needle at the angiocatheter to advance the angiocatheter in the monorail system of the 190 cm EPD filter wire (arrow). (**e**) We inserted a 0.035-inch buddy wire (arrow) into the angiocatheter to navigate the angiocatheter pass through the stent. (**f**) The EPD was successfully removed into the angiocatheter.

In some cases, a 5 Fr Davis angiocatheter (Cook, Bloomington, IN, USA) was used instead of the provided retrieval sheath. As the 190 cm filter wire was too short to introduce the angiocatheter, we created a side hole in the distal segment of the angiocatheter (4–5 cm from the tip) with a 22-gauge needle. This allowed us to advance the angiocatheter into the monorail system without using a docking wire. Although the ledge effect could persist when using the 5 Fr angiocatheter, rotating the curved tip could effectively change the geometry of the filter wire and bypass the impinged segment. When the curved tip of the angiocatheter could not rotate smoothly within the stent, a 0.035-inch Terumo wire (Terumo Corporation, Tokyo, Japan) was used as a buddy wire. The filter was removed into the angiocatheter after passing the angiocatheter through the stent in the remaining six cases (26.1%) (Figure 5).

## 4. Discussion

The main findings of our study were as follows: (1) most technical complications during CAS were associated with use of the EPD, (2) complicated retrieval of the placed filter was the most common adverse event related to the EPD, which is associated with older age, residual stenosis, and use of the Emboshield device, (3) several rescue techniques proposed in the study aided in successful retrieval of the filter, and (4) even when it was associated with distal thrombus migration, rescue techniques were effective in prevention of postprocedural thromboembolic complications.

Retrieval of the filter becomes complicated mainly when the retrieval sheath is trapped by stent struts protruding into the lumen of the vessel. Previous studies have reported the prevalence of difficult EPD retrieval to be between 10.2% and 15.4% [10,11]. Although this prevalence may vary depending on the devices used, the result of our study (14.6%) fell within the range of previous series and proved that it was the most common technical complication during CAS.

Several factors including calcified plaque, the curvature of the stent-placed vessel represented by the TI, and specific types of the used stent, especially the open-cell type of stent, were proposed as potential risk factors [10]. Vessel tortuosity primarily contributes to impingement of the retrieval sheath through the mechanism that the angulated vessel is more likely to cause friction with the intruded stent strut as it advances along the filter wire. In our cohort, there were instances of retrieval sheath trapping at the level of the carotid bulb or the orifice of the stent, where vessel curvature was not severe, but the inner mound was formed by the plaque in the posterior aspect of the carotid bulb. The combination of open-cell-type stents with a larger cell size and a retrieval sheath with a greater ledge effect is known to be a typical circumstance for complicated filter removal [10]. The struts of open-cell-type stents could inwardly protrude in the stenotic segment, which may impede the passage of the retrieval sheath. The outer diameter of the Emboshield retrieval sheath is 0.067 inches, and its relatively large lumen may generate a ledge effect when advanced along a 0.014-inch filter wire. However, considering that complicated filter removal occurred even when using a close-cell-type stent and when using a retrieval sheath with a relatively small outer diameter (e.g., the diameter of the SpiderFX retrieval sheath is 0.054 inches), emphasis should be placed not on the prediction and prevention of adverse events but on the awareness of various rescue techniques for managing these complications effectively. Additionally, densely calcified plaques tend to resist balloon angioplasty, resulting in higher degrees of residual stenosis [12,13]. Considering that trapping usually occurred in the segment where the distance between the filter wire and the stent strut was close enough to cause an impingement, both dense calcification of the plaque and residual stenosis can be considered to increase the risk of complications through similar mechanisms.

Impingement between the stent strut and the retrieval sheath could damage the strut itself and injure the vessel wall or atherosclerotic plaque underneath the stent, which may increase the risk of thromboembolic complications [10]. A previous study reported that a longer procedure time due to complicated filter removal was related to intracranial complications [10]. Furthermore, repeated manipulation of the retrieval sheath, such as a to-and-fro movement, may result in caudal movement of the filter and subsequent entrapment of the filter device in the stent. One study reported a case of a detained protection device during CAS necessitating CEA and emphasized the need to retrieve the filter and employ salvage techniques to prevent unnecessary surgery [14]. Although most cases were successfully managed without neurological sequelae in our series, complicated filter removal was shown to be associated with distal thrombus migration necessitating rescue mechanical thrombectomy. Given that one patient with comorbid flow impairment and distal thrombus migration showed further aggravation of pre-existing neurologic symptoms after successful reperfusion, prompt execution of a series of rescue treatments is essential to weaken the negative effect of complicated filter retrieval on clinical outcomes [5,13,14].

Several studies have reported rescue strategies to retrieve trapped filters. Gentle to-and-fro movements of the retrieval sheath, careful adjustment of the guiding catheter, and external manual compression of the patient’s neck are worth a try as the first attempt to overcome this situation [11,15]. Caution is required because excessive attempts at multiple manipulations of the guiding catheter and retrieval sheath may reduce the stability of the EPD and cause distal embolization. Rescue balloon angioplasty can also induce stent wall apposition and strut remodeling. However, it may not be effective in the absence of residual stenosis or in the presence of dense calcified plaques. Pulling the filter wire to detach it from the stent strut is not recommended because it may result in caudal movement and entrapment of the EPD with the stent.

A guiding catheter with a curved tip was helpful in some instances by altering the relative location of the filter wire to the deployed stent through proximal rotatory manipulation. An 8 Fr Vista Brite tip catheter even with a curved tip possessed a low torque profile; following rotatory movement of the catheter tip, obtaining the correct profile was not plausible in most cases in our cohort. Although the 80 cm Guider Softip was manufactured for coil embolization and possessed the segmental stiffness suitable for proximal to middle ICA placement, it can allow proper torque to change its tip location according to the rotating manipulation of the proximal segment. Although it has the potential risk of sudden drop into the aortic arch during the procedure, we utilized it when navigation of the device was expected to be difficult due to severe angulation in the carotid bulb or due to the presence of densely calcified plaque. Due to the issue of off-label use, there is a definite limitation to its use as a standard tool in every CAS.

The coaxial technique is a standard method to overcome the ledge effect between the catheter and guidewire. Using the coaxial technique with the retrieval sheath is impossible because the microwire must exit through the side hole of the retrieval sheath in the monorail system. A previous study reported overcoming the ledge effect using a balloon catheter, known as the balloon bridge technique [16]. Because a balloon catheter is almost always used during CAS, there is no risk of incompatibility in the system according to the filter wire. For the guiding catheter to pass through the stent while avoiding impingement on the stent strut, a partially inflated balloon was used to cover the large gap between the catheter and filter wire. However, the balloon expanded rapidly when it first began to inflate owing to the elastic hysteresis, and the diameter of the balloon is usually smaller than 2 cm to be able to eliminate the gap. The compliance chart of the balloon catheter used in our case (Sterling, Boston Scientific, Natick, MA, USA) did not provide information on the balloon diameter below its nominal inflation pressure. Although a previous report introduced an adjunctive technique in which the balloon was inflated outside the stent and concurrently advanced into the stent while maintaining inflation, we first performed rescue angioplasty and then adjusted the diameter of the balloon by deflating it [16].

Another rescue technique alternatively utilized a 5 Fr angiocatheter with a curved tip as a retrieval sheath. In some instances, the balloon catheter could not pass through the proximal marker of the stent, which did not allow for the use of repetitive angioplasty or the balloon bridge technique. In this situation, the method of bypassing the stenotic segment using the highly controllable angiocatheter instead of the retrieval sheath can be very useful. Several studies have introduced the use of a curved 5 Fr catheter with a docking wire instead of a retrieval sheath [5,17]. Because the 190 cm long wire of the installed filter is not sufficient to insert a 125 cm 5 Fr catheter, a docking wire can be used to extend the length of the microwire in this situation [18]. In our cases, we made a side hole to pull out the filter wire, and a 5 Fr catheter was used as the retrieval sheath. If the catheter could not advance or rotate smoothly in the stent, the introduction of a buddy wire was helpful.

The limitations of our study mainly stemmed from its single-center observational design with a small number of patients, resulting in limited use of the device. As we predominantly used an open-cell-type stent, the overall incidence of difficult removal may be higher than when using closed-cell-type stents. Further, although complicated filter retrieval was mostly observed in patients using the Emboshield device in our study, the relatively small number of patients using SpiderFX as controls hinders meaningful conclusions from being drawn based on our cohort. Given that an analysis of other types of EPDs (e.g., RX accunet, Angioguard, Filterwire) was not conducted, further studies with larger cohorts using various combinations of devices, including different filter types, stent designs, and guiding catheters, are warranted. Because of the retrospective design of the study, there may be selection bias, such as patients with a complex anatomy may not under CAS. Finally, we included both symptomatic and asymptomatic patients, as well as patients with acute tandem occlusion. The incidence of thromboembolic complication such as minor and major infarctions may differ based on the patients’ baseline characteristics; our study showed exaggerated incidence rates specifically for major infarction compared to previous studies with low complication rates [19,20]. Most patients with new lesions on DWI showed no neurologic symptoms in our study; however, the incidence was also higher than in previous studies [21,22]. For patients with acute infarction who showed worsening of the NIHSS score, it was difficult to distinguish whether the cause was progression of the pre-existing infarction or thromboembolism following the procedure. Nonetheless, complicated filter retrieval can occur at any time during procedures using EPDs regardless of the patients’ clinical setting, which can lead to transient but potentially harmful effects on cerebral perfusion. Therefore, establishing a stepwise strategy for rescue management and promptly applying it in sequence to resolve the issue is crucial.

## 5. Conclusions

Complicated filter retrieval of EPDs frequently occurs during CAS when carotid stenosis is resistant to balloon angioplasty, particularly when a specific combination of device provokes inward protrusion of the stent strut and a substantial ledge effect between the filter wire and retrieval sheath. Although it was not associated with the occurrence of postprocedural neurologic deterioration, it can induce intraprocedural thrombus migration into the intracranial vessels, necessitating adjunctive mechanical thrombectomy. Therefore, being well acquainted with various rescue techniques is crucial to increasing technical success rates and ensuring safety during CAS.

## Figures and Tables

**Table 1:** Demographics, baseline clinical and angiographic data, used devices for carotid artery stenting, and periprocedural complications of enrolled 158 patients.

Characteristics	Numbers (%) or Average ± 2 SD
Age (years)	71.3 ± 9.4
Male	136 (86.1%)
Right side	86 (54.4%)
Symptomatic	97 (61.4%)
Underlying disease	
Hypertension	99 (62.7%)
Diabetes	59 (37.3%)
Dyslipidemia	38 (24.1%)
Current smoker	21 (13.3%)
Previous cardiovascular disease	27 (17.1%)
Antiplatelet therapy before stenting	137 (86.7%)
Emergency stenting	28 (17.7%)
Stenotic degree	
50~70%	20 (12.7%)
70~99%	102 (64.5%)
Near occlusion	21 (13.3%)
Complete occlusion	15 (9.5%)
Contralateral occlusion	11 (7.4%)
Tortuosity index (°)	43.5 ± 26.9
Type of guiding catheter	
Vista Brite Tip	110 (69.6%)
Softip Guider	25 (15.9%)
Balloon guiding catheter	19 (12.0%)
Shuttle	4 (2.5%)
Type of embolic protection device	
Emboshield	119 (75.3%)
Spider	39 (24.7%)
Types of stents	
Open cell	139 (88.0%)
Closed cell	19 (12.0%)
Residual stenosis (%)	15.2 ± 15.2
Intraprocedural complications	40 (25.3%)
Postprocedural thromboembolic complications with minor infarction	46 (29.1%)
Postprocedural thromboembolic complications with major infarction	3 (1.9%)
Postprocedural hemorrhagic complications	1 (1.6%)
Mortality within 3 months	0 (0%)

**Table 2:** The types and incidences of the technical complications during carotid artery stenting.

Types of Intracranial Complications	Number of Occurrences
Balloon angioplasty-related adverse events	2
Severe vasovagal reaction	1
Arterial dissection	1
Perforation	0
Embolic protection device-related adverse events	37
Unsuccessful deployment of the filter	0
Filter wedging into the stent delivery catheter tip or stent	0
Arterial dissection	0
Complicated filter retrieval	18
Flow impairment due to clogging emboli at the filter	11
Distal thrombus migration after filter removal	3
Complicated filter retrieval + flow impairment	2
Complicated filter retrieval + distal thrombus migration	2
Complicated filter retrieval + flow impairment + distal thrombus migration	1
Stenting-related adverse events	1
Stent migration	1
In-stent thrombus	0
Total number of intraprocedural complications	40

**Table 3:** Risk factors and clinical implications of complicated filter retrieval.

	Smooth Filter Retrieval (*n* = 135)	Complicated Filter Retrieval (*n* = 23)	*p* Value
Age (years)	70.6 ± 9.7	75.5 ± 6.7	0.022
Male	119 (88.1%)	17 (73.9%)	0.068
Right side	74 (54.8%)	12 (52.2%)	0.814
Symptomatic	80 (59.3%)	16 (69.6%)	0.489
Underlying disease			
Hypertension	84 (62.2%)	15 (65.2%)	1.000
Diabetes	46 (34.1%)	13 (56.5%)	0.060
Dyslipidemia	31 (23.0%)	7 (30.4%)	0.437
Current smoker	19 (14.1%)	2 (8.7%)	0.741
Previous cardiovascular disease	25 (18.5%)	2 (8.7%)	0.371
Antiplatelet therapy before stenting	116 (85.9%)	21 (91.3%)	0.741
Emergency stenting	26 (19.3%)	3 (13.0%)	0.574
Stenotic degree			0.634
50~70%	16 (11.9%)	4 (17.4%)	
70~99%	86 (63.7%)	16 (69.6%)	
Near occlusion	20 (14.8%)	1 (4.3%)	
Complete occlusion	13 (9.6%)	2 (8.7%)	
Contralateral occlusion	9 (6.7%)	2 (8.7%)	0.663
Tortuosity index (°)	42.2 ± 25.2	51.3 ± 35.1	0.132
Type of guiding catheter			0.254
Vista Brite Tip	97 (71.8%)	13 (56.5%)	
Softip Guider	18 (13.3%)	7 (30.4%)	
Balloon guiding catheter	16 (11.9%)	3 (13.0%)	
Shuttle	4 (3.0%)	0 (0.0%)	
Type of embolic protection device			0.016
Emboshield	97 (71.9%)	22 (95.7%)	
Spider	38 (28.1%)	1 (4.3%)	
Types of stents			1.000
Open cell	118 (87.4%)	21 (91.3%)	
Closed cell	17 (12.6%)	2 (8.7%)	
Residual stenosis (%)	13.7 ± 14.5	24.2 ± 16.4	0.002
Distal thrombus migration	3 (2.2%)	3 (13.0%)	0.041
Minor infarction	37 (27.4%)	9 (39.1%)	0.320
Major infarction	2 (1.5%)	1 (4.3%)	0.378
Intracranial hemorrhage	1 (0.7%)	0 (0%)	1.000
Mortality	0 (0%)	0 (0%)	N/A

N/A: not accessed.

## Data Availability

All data generated and analyzed during this study are included in the published article.

## Supplementary Materials

The following supporting information can be downloaded at: https://www.mdpi.com/article/10.3390/diagnostics14232622/s1, Supplementary Figure S1: Measurement of tortuosity index; Supplementary Table S1: Detailed clinical, angiographic, and procedural data of the patients with complicated filter retrieval were summarized. Comorbid adverse event, rescue treatment, and clinical outcomes were also described.

## Abbreviations

CAS	carotid artery stenting
CCA	common carotid artery
CEA	carotid endarterectomy
EPD	embolic protection device
ICA	internal carotid artery
NIHSS	National Institutes of Health Stroke Scale
