# Supplementary material for: Technical Complications Associated with Embolic Protection Device During Carotid Artery Stenting: Incidence, Risk Factors, Clinical Implications, and Rescue Maneuvers
Source: Diagnostics (Basel). 2024 Nov 21;14(23):2622. doi: 10.3390/diagnostics14232622 (PMC11640039; doi:10.3390/diagnostics14232622)
Supplement: Supplementary file 1 [file diagnostics-14-02622-s001.zip › Supplementary Table S1.pdf]

Supplementary Table S1. Detailed clinical, angiographic, and procedural data of the patients with complicated filter retrieval were summarized. Comorbid adverse event, rescue treatment, and clinical outcomes were also described.

No.	Sex/ Age	Filter	Stent	Indication	TI	Residual stenosis	Rescue technique	Comorbid adverse event	Management	Thromboembolic complications
1	M/84	Emboshield	Acculink	Symptomatic severe stenosis	121.2	37.9	Balloon bridge technique	Flow impairment due to captured emboli	Flow restoration after EPD removal	Minor infarction
2	M/78	Emboshield	Acculink	Symptomatic severe stenosis	64.6	0	Balloon bridge technique			None
3	M/71	Emboshield	Acculink	Symptomatic severe stenosis	96.7	39.3	Balloon bridge technique	Distal thrombus migration to M2	Contact aspiration (mTICI 3)	Minor infarction
4	M/76	Emboshield	Acculink	Asymptomatic severe stenosis	31.1	13.3	Swallowing, neck rotation, to-and-fro movement			None
5	M/69	Emboshield	Acculink	Asymptomatic severe stenosis	20.8	0	Balloon bridge technique			None
6	M/66	Emboshield	Acculink	Isolated acute ICA occlusion (Emergency)	30.6	38.8	Swallowing, neck rotation, to-and-fro movement	Distal thrombus migration to M1 & Flow impairment due to captured emboli	Contact aspiration (mTICI 3)	Major stroke (Aggravation of neurologic symptom)
7	M/73	Emboshield	Protégé	Symptomatic severe stenosis	40.1	7	Balloon bridge technique			None
8	M/90	Spider	Protégé	Isolated acute ICA occlusion (Emergency)	58.8	22.9	Swallowing, neck rotation, to-and-fro movement			No symptom change
9	F/72	Emboshield	Protégé	Symptomatic severe stenosis	15.2	0	Balloon bridge technique			None
10	F/80	Emboshield	Acculink	Symptomatic near occlusion	28.6	21.9	Swallowing, neck rotation, to-and-fro movement			None
11	M/72	Emboshield	Acculink	Symptomatic severe stenosis	33.3	32.2	5 F Davis catheter with buddy wire			None
12	M/85	Emboshield	Protégé	Asymptomatic severe stenosis	106.8	34.8	Curved tip guiding catheter			Minor infarction
13	F/69	Emboshield	Acculink	Asymptomatic	23.1	37.8	Swallowing, neck			None

				severe stenosis			rotation, to-and-fro movement			
14	F/68	Emboshield	Precise	Symptomatic severe stenosis	144.9	47.4	5 F Davis catheter with buddy wire			None
15	M/67	Emboshield	Carotid Wallstent	Tandem occlusion in ICA and M1 (Emergency)	26.3	39.2	5 F Davis catheter	Distal thrombus migration to ICA terminus	Stent retriever thrombectomy (mTICI 2b)	Minor infarction (Slight aggravation of symptom)
16	F/69	Emboshield	Precise	Asymptomatic severe stenosis	35.5	0	Swallowing, neck rotation, to-and-fro movement			None
17	M/77	Emboshield	Precise	Symptomatic severe stenosis	66.8	28.7	Swallowing, neck rotation, to-and-fro movement	Flow impairment due to captured emboli	Flow restoration after EPD removal	Minor infarction
18	M/79	Emboshield	Protégé	Symptomatic moderate stenosis	53.0	14.7	Swallowing, neck rotation, to-and-fro movement			None
19	M/78	Emboshield	Protégé	Symptomatic severe stenosis	20.5	42.9	5 F Davis catheter			None
20	M/73	Emboshield	Acculink	Asymptomatic severe stenosis	26.4	37.6	Swallowing, neck rotation, to-and-fro movement			Minor infarction
21	M/75	Emboshield	Protégé	Symptomatic severe stenosis	39.3	22.5	5 F Davis catheter			Minor infarction
22	F/88	Emboshield	Carotid Wallstent	Symptomatic moderate stenosis	34.0	0	Balloon bridge technique			Minor infarction
23	M/77	Emboshield	Protégé	Asymptomatic severe stenosis	62.96	36.6	5 F Davis catheter			None

DWI, diffusion-weighted imaging; EPD, Embolic protection device; ICA, internal carotid artery; M1, middle cerebral artery M1 segment; M2, middle cerebral artery M2 segment; mTICI, modified treatment for cerebral ischemia
