# Supplementary figures and images for: Technical Complications Associated with Embolic Protection Device During Carotid Artery Stenting: Incidence, Risk Factors, Clinical Implications, and Rescue Maneuvers
Source: Diagnostics (Basel). 2024 Nov 21;14(23):2622. doi: 10.3390/diagnostics14232622 (PMC11640039; doi:10.3390/diagnostics14232622)

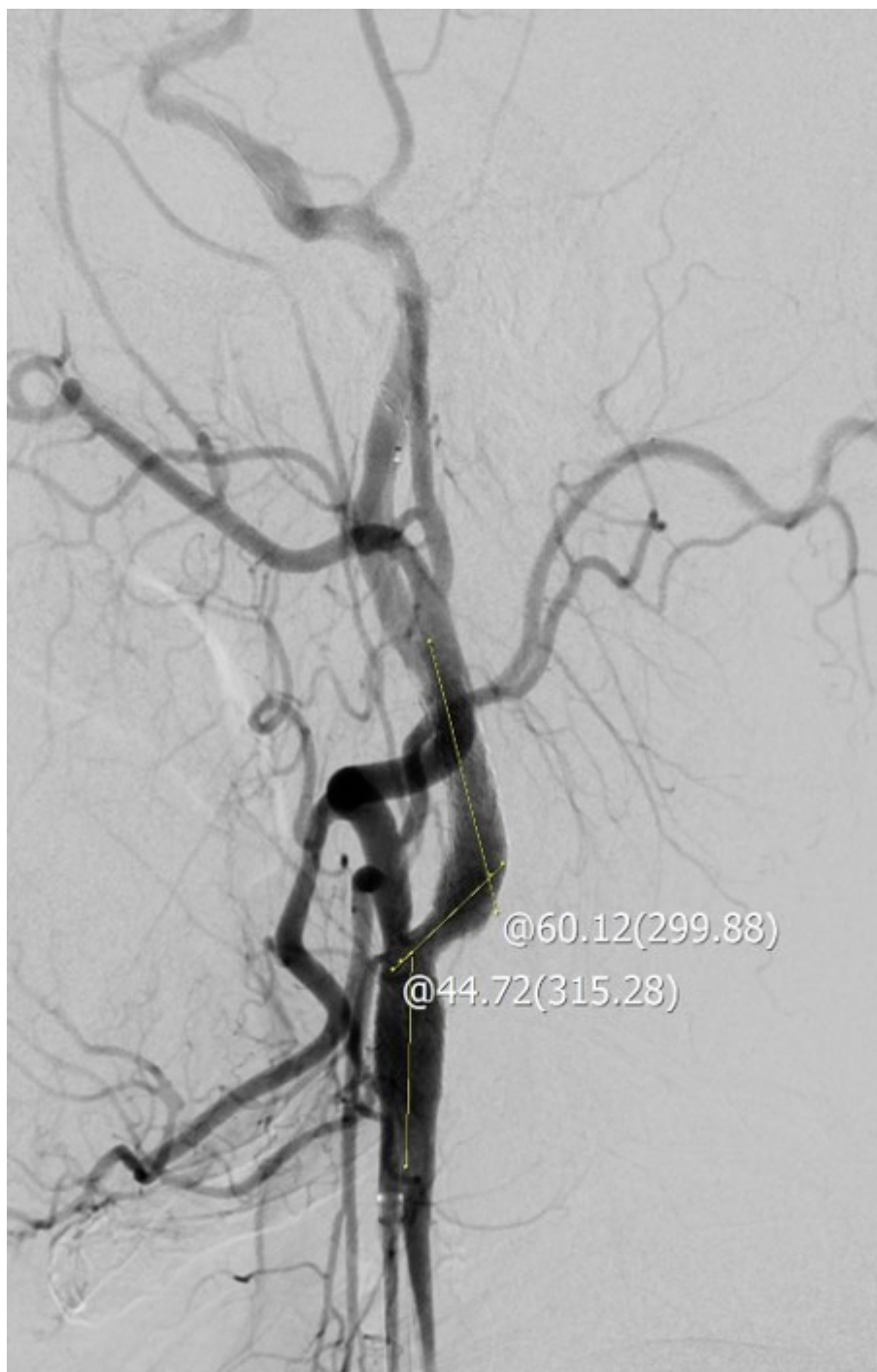

**Supplementary Figure S1.** Measurement of tortuosity index.

Supplement: Supplementary file 1 [file diagnostics-14-02622-s001.zip › Supplementary Figure S1.pdf]
